# Supplementary figures and images for: UltraTimTrack: a Kalman-filter-based algorithm to track muscle fascicles in ultrasound image sequences
Source: PeerJ Comput Sci. 2025 Jan 24;11:e2636. doi: 10.7717/peerj-cs.2636 (PMC11784871; doi:10.7717/peerj-cs.2636)

# Sinusoidal trials

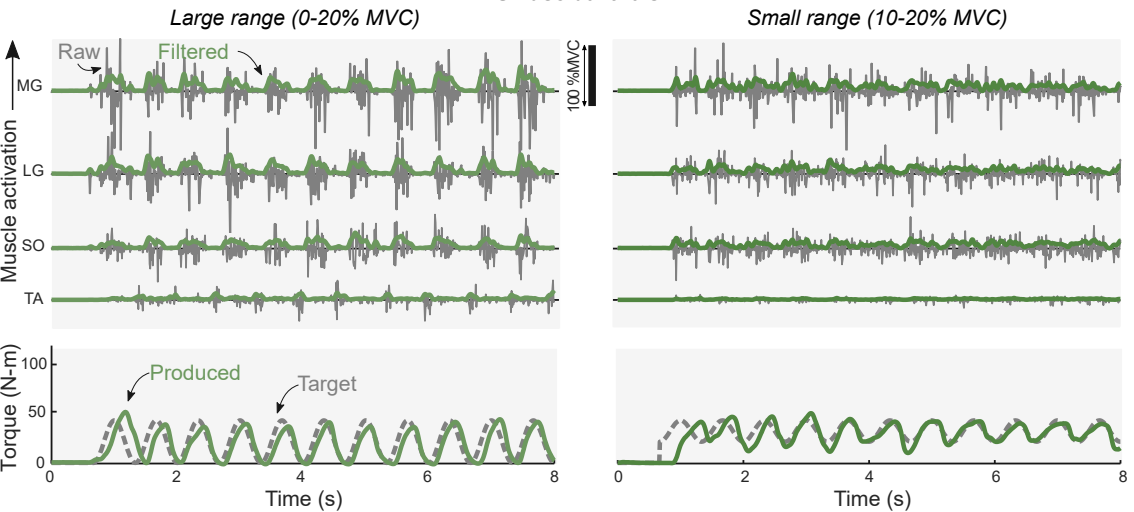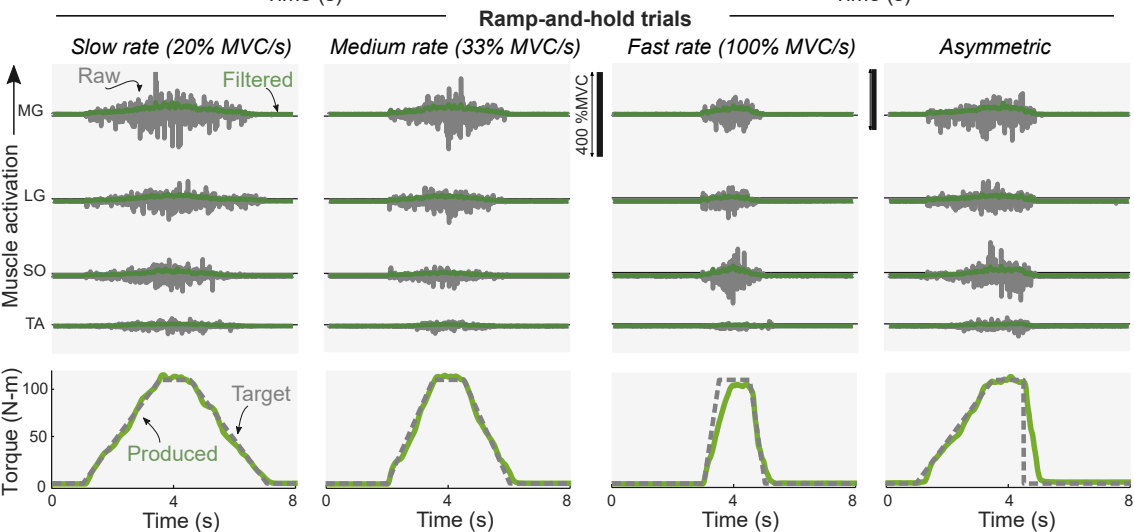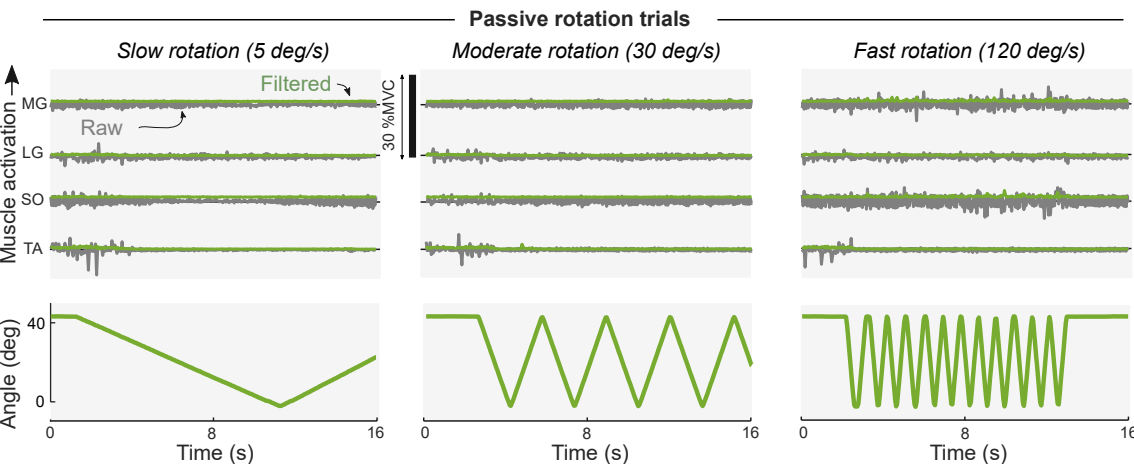

Supplement: Figure S1 — Muscle activity levels of medial gastrocnemius (MG), lateral gastrocnemius (LG), soleus (SO) and tibialis anterior (TA) are shown either as raw (grey) or filtered (green) electromyography signals. MVC: Maximal Voluntary Contraction. [file peerj-cs-11-2636-s001.pdf]

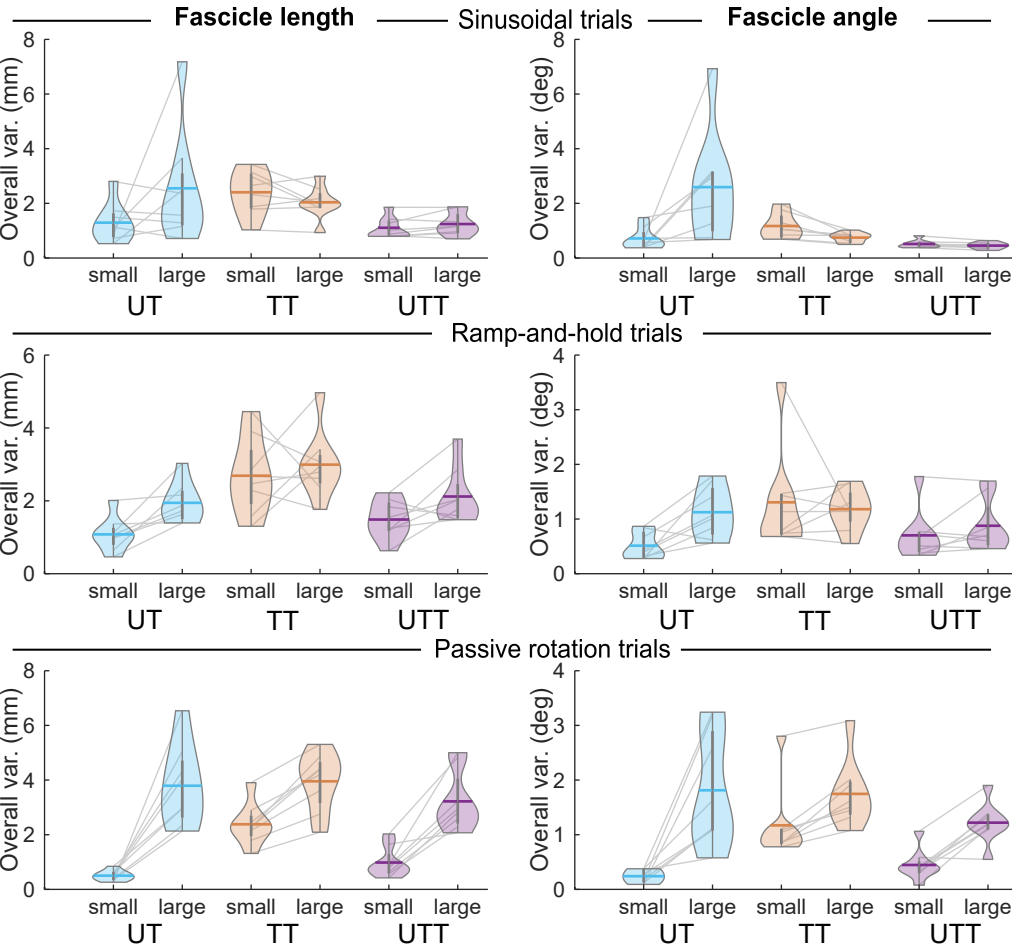

Supplement: Figure S2 — Overall variability (var.) of fascicle length (left) and fascicle angle (right) estimates of UltraTrack (UT, blue), TimTrack (TT, red) and UltraTimTrack (UTT, purple). Top row: For sinusoidal trials, small and large image-to-image dissimilarity corresponds to small and large torque range, respectively. Middle row: For ramp-and-hold trials, small and large image-to-image dissimilarity corresponds to slow and fast ramp rates, respectively. Bottom row: For passive rotation trials, small and large image-to-image dissimilarity corresponds to slow and fast rotation rates, respectively. [file peerj-cs-11-2636-s002.pdf]
