# Supplementary material for: UltraTimTrack: a Kalman-filter-based algorithm to track muscle fascicles in ultrasound image sequences
Source: PeerJ Comput Sci. 2025 Jan 24;11:e2636. doi: 10.7717/peerj-cs.2636 (PMC11784871; doi:10.7717/peerj-cs.2636)
Supplement: Table S1 [file peerj-cs-11-2636-s003.docx]

|  | Mean torque  (% MVC) | Mean MG EMG  (% MVC) | Mean LG EMG  (% MVC) | Mean SO EMG  (% MVC) | Mean TA EMG  (% MVC) |
| --- | --- | --- | --- | --- | --- |
| Ramp-and-hold trials – Low image quality | | | | | |
| Slow | 21.1 ± 1.2 | 13.3 ± 4.8 | 8.1 ± 3.3 | 11.2 ± 3.0 | 2.2 ± 1.4 |
| Medium | 15.1 ± 0.7 | 9.2 ± 2.8 | 6.0 ± 2.8 | 8.9 ± 2.8 | 1.8 ± 1.0 |
| Fast | 9.3 ± 0.6 | 6.8 ± 2.8 | 4.4 ± 2.1 | 6.0 ± 1.3 | 1.5 ± 0.9 |
| Asymmetric | 14.4 ± 0.8 | 9.4 ± 3.3 | 6.3 ± 2.9 | 8.0 ± 2.4 | 1.8 ± 1.1 |
|  |  |  |  |  |  |
| Ramp-and-hold trials – High image quality | | | | | |
| Slow | 20.9 ± 1.1 | 13.1 ± 4.4 | 8.1 ± 3.8 | 11.0 ± 2.7 | 2.1 ± 1.3 |
| Medium | 15.1 ± 0.8 | 9.6 ± 3.2 | 5.8 ± 2.8 | 8.3 ± 2.2 | 1.7 ± 1.0 |
| Fast | 9.1 ± 0.6 | 6.8 ± 2.9 | 4.4 ± 2.0 | 6.1 ± 1.7 | 1.4 ± 0.8 |
| Asymmetric | 14.5 ± 1.0 | 9.8 ± 3.5 | 6.5 ± 3.0 | 8.1 ± 2.4 | 1.9 ± 1.0 |
|  |  |  |  |  |  |
| Passive trials | | | | | |
| Slow | NA | 0.7 ± 0.6 | 0.8 ± 0.9 | 1.0 ± 0.4 | 0.7 ± 0.5 |
| Medium | NA | 0.9 ± 0.9 | 0.8 ± 0.8 | 1.0 ± 0.4 | 0.7 ± 0.5 |
| Fast | NA | 1.2 ± 0.8 | 1.2 ± 0.9 | 1.8 ± 1.1 | 0.7 ± 0.4 |
|  |  |  |  |  |  |
| Sinusoidal trials | | | | | |
| 0-20% MVC | 9.8 ± 0.9 | 11.7 ± 4.2 | 6.9 ± 3.8 | 9.8 ± 2.3 | 2.3 ± 1.5 |
| 10-20% MVC | 14.6 ± 0.9 | 13.2 ± 4.7 | 6.7 ± 3.3 | 9.9 ± 3.8 | 1.6 ± 0.8 |
