# Supplementary material for: UltraTimTrack: a Kalman-filter-based algorithm to track muscle fascicles in ultrasound image sequences
Source: PeerJ Comput Sci. 2025 Jan 24;11:e2636. doi: 10.7717/peerj-cs.2636 (PMC11784871; doi:10.7717/peerj-cs.2636)
Supplement: Article S1 — Summary of Kalman filter equations employed by the proposed UltraTimTrack algorithm. [file peerj-cs-11-2636-s008.docx]

# Supplemental Article S1

A Kalman filter considers the state (vector) $z$ of a dynamical system at time $i$. It assumes that the current state (vector) $z_{i}$ depends on state-transition model $A$, control-input model $B$, the previous state (vector) $z_{i-1}$, the current input $u_{i}$, and the current process noise $w_{i}$:

$z_{i}=A\cdot z_{i-1}+B\cdot u_{i}+w_{i}$ Eq. S1

Process noise $w_{i}$ is unknown, and assumed to be drawn from a zero-mean normal distribution with covariance $Q_{i}$. Because process noise $w_{i}$ is unknown, state $z_{i}$ cannot be computed directly. Instead, the Kalman filter can predict state $z_{i}$, and update this prediction using a measurement $\tilde{z}_{i}$ to yield an optimal state estimate $\hat{z}_{i}$.

First, an *a-priori* state estimate ${z_{i}}^{-}$ is predicted based on the previous state estimate $\hat{z}_{i-1}$:

${z_{i}}^{-}=A\cdot\hat{z}_{i-1}+B\cdot u_{i}$ Eq. S2

Next, the *a-priori* state estimate ${z_{i}}^{-}$ is updated using a measurement $\tilde{z}_{i}$ to yield an optimal *a-posteriori* state estimate $\hat{z}_{i}$:

$\hat{z}_{i}={z_{i}}^{-}+K_{i}\cdot({z_{i}}^{-}-\tilde{z}_{i})$ Eq. S3

Here, $K_{i}$ is the Kalman gain, and $\tilde{z}_{i}$ is a measurement that relates to state $z_{i}$, through measurement model $H$, and measurement noise $v_{i}$:

$\tilde{z}_{i}={H\cdot z}_{i}+v_{i}$ Eq. S4

Measurement noise $v_{i}$ is unknown, and assumed to be drawn from a zero-mean normal distribution with covariance $R_{i}$. Because measurement noise $v_{i}$ is unknown, determining the optimal value of Kalman gain $K_{i}$ is not trivial. To inform Kalman gain $K_{i}$, the Kalman filter also predicts the state covariance $P_{i}$. First, the *a-priori* state covariance estimate ${P_{i}}^{-}$ is predicted based on the previous state covariance $\hat{P}_{i-1}$:

${P_{i}}^{-}=A\cdot\hat{P}_{i-1}\cdot A^{T}+Q_{i}$ Eq. S5

Next, the Kalman gain $K_{i}$ is computed as:

$K_{i}={P_{i}}^{-}\cdot H^{T}\cdot\left( H\cdot{P_{i}}^{-}\cdot H^{T}+R_{i} \right)^{-1}$ Eq. S6

The reader is referred to Kalman (1960) for derivation and details. Once the Kalman gain is known, the *a-posteriori* state estimate $\hat{z}_{i}$ can be computed (Eq. S3), as well as its covariance $\hat{P}_{i}$:

$\hat{P}_{i}=(I-K_{i}\cdot H)\cdot{P_{i}}^{-}$ Eq. S7

Here, $I$ is the identity matrix.

After Kalman filtering, *a-posteriori* state estimate $\hat{z}_{i}$ can be smoothed using a Rauch-Tung-Striebel smoother. This smoother works in the backward direction: it computes the current smoothed state estimate $\check{z}_{i}$ from the next smoothed state estimate $\check{z}_{i+1}$, and the next *a-priori* state estimate ${z_{i+1}}^{-}$:

$\check{z}_{i}=\hat{z}_{i}+C_{i}\cdot(\check{z}_{i+1}-{z_{i+1}}^{-})$ Eq. S8

Here, $C_{i}$ is the smoothing gain, which is computed from the next *a-priori* state covariance ${P_{i+1}}^{-}$ and the current *a-posteriori* state covariance $\hat{P}_{i}$:

$C_{i}= \hat{P}_{i}\cdot A^{T}\cdot{{{(P}_{i+1}}^{-})}^{-1}$ Eq. S9

The reader is referred to Rauch et al. (1965) for derivation and details.

**References**

Kalman RE. 1960. A New Approach to Linear Filtering and Prediction Problems. *Journal of Basic Engineering* 82:35–45. DOI: 10.1115/1.3662552.

Rauch HE, Striebel CT, Tung F. 1965. Maximum likelihood estimates of linear dynamic systems. *American Institute of Aeronautics and Astronautics Journal* 3:1445-1450. DOI: 735 10.2514/3.3166.
